# Exploring trial publication and research waste in COVID-19 randomised trials of hydroxychloroquine, corticosteroids, and vitamin D: a meta-epidemiological cohort study

**DOI:** 10.1186/s12874-023-02110-4

**Published:** 2024-01-23

**Authors:** Lisa Fincham, Ameer Hohlfeld, Mike Clarke, Tamara Kredo, Michael McCaul

**Affiliations:** 1 Division of Epidemiology and Biostatistics, Department of Global Health, Faculty of Medicine and Health Sciences, Stellenbosch University, Francie van Zijl Drive, Tygerberg, Cape Town, 7505 South Africa; 2 Health Systems Research Unit, South African Medical Research Council, Cape Town, South Africa; 3 Northern Ireland Methodology Hub, Centre for Public Health, Queen’s University Belfast, Belfast, Northern Ireland; 4 Division of Clinical Pharmacology, Department of Medicine and Division of Epidemiology and Biostatistics, Department of Global Health, Faculty of Medicine and Health Sciences, Stellenbosch University, Cape Town, South Africa

**Keywords:** Research waste, Publication bias, Randomised trial, COVID-19, Methods research

## Abstract

**Background:**

The global research response to the COVID-19 pandemic was impressive, but also led to an infodemic and considerable research waste. Registered, but unpublished trials added to this noise. We aimed to determine the proportion of registered randomised trials of common COVID-19 treatments that were published and to describe the characteristics of these trials to examine the association between trial characteristics, publication status and research waste.

**Methods:**

This meta-epidemiological cohort study used a sample of randomised trials of corticosteroids, hydroxychloroquine or vitamin D as treatments for COVID-19, registered between 1 November 2019 and 31 December 2021 and available via the WHO ICTRP portal. We searched for the trials’ published results up to 20 October 2022. We extracted the trial characteristics, analysing with descriptive statistics. We performed univariate logistic regression to examine the association between trials’ characteristics and publication status, followed by multiple logistic regression using significant characteristics to assess the association between trial characteristics and publication status.

**Results:**

We identified 357 eligible trials on ICTRP. Of these, 107 (30%) had published or made their results available publicly by 20 October 2022, while 250 (70%) had not been published or shared their results publicly. Multiple logistic regression analysis showed that a larger target sample size was a significant positive predictor of publication with target sample sizes above 300 almost tripling the odds of publication (aOR: 2.75, 95% CI: 1.35 to 5.62).

**Conclusions:**

Less than one third of registered trials made their results public and our findings identified that many trialists had not updated their trial registry entry with the trial status, results or both. Failure to share trial results publicly is a disservice to patients, clinicians and policy makers and adds to research waste.

**Supplementary Information:**

The online version contains supplementary material available at 10.1186/s12874-023-02110-4.

## Introduction

In response to the COVID-19 pandemic, researchers initiated thousands of randomised trials seeking possible treatments. Many of these trials have been registered, conducted and published but the amount of research and the speed at which it was produced created an infodemic making it difficult for potential users of the research to discern what information is relevant, accurate and current [1]. Furthermore, the rush to conduct trials meant that many had methodological shortcomings, such as small sample sizes and lack of blinding or allocation concealment [2]. This, coupled with unnecessary duplication, has led to a high “noise to signal” ratio in the COVID-19 evidence base and research waste [3].

Research waste can occur during any stage of the research process [4]. In the production phase of research, it occurs when the question is irrelevant to clinicians or patients or has already been answered definitively. Chalmers et al. [5] highlighted that new research should not be started unless existing research cannot answer the question adequately and systematic reviews are one way to determine this [6]. For example, through 2020 and 2021, several trials of chloroquine or hydroxychloroquine (HCQ) for treating COVID-19 were published, and a Cochrane systematic review in February 2021 concluded that HCQ has little to no effect on the risk of death and that further trials of HCQ or chloroquine should not be carried out [7–10]. Further, research waste can arise because a lack of coordination and collaboration can lead to unnecessary duplication of research which will add limited evidence strength. For example, even though larger, multisite studies recruiting thousands of patients might be underway, multiple small single-site studies still often take place. This can create waste [2]. In the reporting phase of research, waste is generated when published studies have unusable or biased results. Under-reported or unpublished research also contribute to research waste and breach the researcher’s ethical obligation to make results of research on humans publicly available [5, 11].

Research waste stems from research with little to no societal, educational or stakeholder benefit [4]. Poorly reported or unpublished results compound research waste, pose a risk to the care of future patients and present ethical concerns [5, 12]. Although research waste is not a new problem, it was accelerated by the COVID-19 pandemic and was prevalent across trials, evidence synthesis and guidelines [2, 13, 14].

Publication of results helps limit research waste by ensuring that studies add to the knowledge pool regardless of their results [15]. Traditionally, publication was achieved by publishing in a peer-reviewed journal but many trial registries, which help to improve transparency about trial methods, also now provide a facility for researchers to upload their results [16]. Trial registries that meet the requirements of the World Health Organization (WHO) and International Committee of Medical Journal Editors (ICMJE), are searchable through the International Clinical Trial Registry Platform (ICTRP), allowing access to information on hundreds of thousands of randomised trials [17]. It has long been known that the results of trials influence whether or not they are published but the information available in trial registries can also be used to investigate whether any design characteristics of a trial are associated with the publication of its results [18].

The large number of registered trials of treatments for COVID-19, the global nature of the research and the speed at which research took place, provide an opportunity to assess trial characteristics that may lead to successful publication in peer-reviewed journals. Therefore, we conducted this meta-epidemiological cohort study to determine the proportion of registered randomised trials of COVID-19 treatment that were published in peer-reviewed journals, or on preprint servers or shared their results publicly in other ways; to describe the characteristics of these trials across three possible treatments (corticosteroids, hydroxychloroquine and vitamin D); and to examine the association between trial characteristics, publication status and research waste.

## Methods

We conducted a meta-epidemiological cohort study of randomised trials of hydroxychloroquine, corticosteroids and vitamin D, identified on ICTRP that were registered up to 31 December 2021. These three possible treatments were chosen to limit the scope while still exploring a variety of research, allowing us to include a treatment for which there is now a high certainty of evidence of no mortality benefit, hydroxychloroquine (both hydroxychloroquine and chloroquine were considered as one treatment form); a treatment for which there is high certainty of evidence of a mortality benefit, corticosteroids; and a treatment for which the effects are still uncertain and controversial, Vitamin D (both Ergocalciferol and Cholecalciferol were considered as one treatment form) [10, 19, 20]. We also assessed how many trials had been registered after key systematic reviews had been published which provided high certainty evidence of the effects of the treatment on mortality. We used an *a priori* protocol (which was established before the data search was done and submitted to the Stellenbosch University Health Research Ethics Committee and received ethics exemption: X22/09/003_COVID-19) and used a flow diagram to present our findings which is based on the PRISMA guidance [21].

### Search methods and screening

We searched ICTRP for randomised trials of treatments for COVID-19 which had been registered between 1 November 2019 and 31 December 2021. A separate search was conducted on ICTRP for each of the three treatment groups using a combination of keywords and free text words (Additional file 1). The searches were restricted to COVID-19 using ICTRP’s preloaded filter and all trial phases were included. No other filters or eligibility criteria were applied in the search. The search was completed on 13 September 2022, allowing eight months from the end of the registration date eligibility criterion for the transfer of information to ICTRP. Results were downloaded into a Microsoft Excel spreadsheet for screening. During screening, eligibility criteria were applied by one author (LF) for final inclusion.

### Eligibility criteria

We included registered trials if they met the following criteria: [1] registration (prospective or retrospective) on the trial registry before 31 December 2021; [2] the primary purpose of the trial was treatment of COVID-19; [3] study design was a randomised trial; and [4] the target intervention in the trial was (or included) one or more of the following: hydroxychloroquine, corticosteroids or vitamin D. We did not limit by country, language or treatment setting. We excluded registered trials if [1] the primary purpose was supportive care or prevention; [2] it was quasi-randomised or not randomised; and [3] it was not performed in humans.

### Data extraction

All available trial characteristics were extracted from the ICTRP data (Additional file 2). Our primary outcome of interest was the proportion of registered trials that were published, or had reported their results publicly in another way, but publication status was not an available field in the ICTRP data. Consequently, a public platform search was conducted to follow up each trial in this cohort. The search was done on PubMed and Google Scholar between 10 and 20 October 2022 using primarily the trial identifier. When this search did not yield a record, the registered title was used in the search, and retrieved records were checked against the authors and the country of origin. If we found the results of the trial in the public domain, we regarded it as “published”. If it was not found, it was classified as “not published” and did not contact those responsible for registering the trial for further information. We categorised publication status as follows: [1] published in a peer-reviewed journal, [2] published on a pre-print server, [3] mention of available results on ICTRP, and [4] other (such as a research letter or retraction). When the search found more than one such record for a trial, we used the most up-to-date record. For example, if a trial was published in a peer-reviewed journal and then later retracted, we counted the retracted record. Current trial status was not available in the ICTRP data, so we searched for this information in the relevant primary registry for each included trial and categorised it as unknown, ongoing, complete and terminated/suspended. The ongoing category included trials that were yet to start recruiting, busy recruiting, active or ongoing according to the primary trial registry. We translated the necessary information from the primary trial registries if it was not in English.

### Data management and coding of trial characteristics

The characteristics’ terminology was largely based on the ICTRP terminology, including phase of trials. Although ICTRP, and many of the primary trial registries, have a primary sponsor field, they do not explicitly state who funded the trial or the source of any financial support. The primary sponsor is defined as the individual or organisation that takes responsibility for the initiation and management of the trial, which may include financial responsibility [22]. Based on their main purpose or the description on their website, we divided the primary sponsors into the following groups: [1] academic, [2] research institute, [3] government, [4] medical centre, [5] pharmaceutical company, [6] organisation, [7] other and no sponsor.

### Data analysis

We conducted a descriptive analysis of the trial characteristics in STATA 16. We explored the association of individual trial characteristics with publication status using a univariate regression analysis, presenting odds ratios (OR) and confidence intervals (CI). When each level of publication status was compared across the three treatments, we found that the results were homogenous with overlapping confidence intervals and therefore pooled the data from all three treatments to maximise power in the regression models. We further investigated the relationship between trial characteristics and publication status using a multivariable logistic regression model. Trial characteristics associated with publication status in the univariate test (p ≤ 0.2) were included in the logistic regression model, for which *P*-values ≤ 0.05 were deemed statistically significant for the final model.

## Results

### Search results

**Fig. 1:**
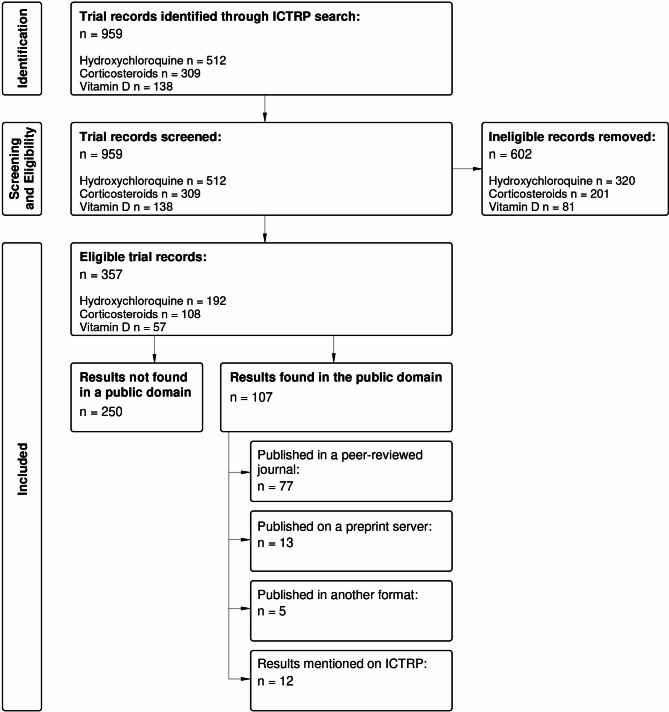
Study flow diagram

Our ICTRP search for trials of hydroxychloroquine, corticosteroids or vitamin D for COVID-19 yielded 959 trial registrations on 13 September 2022. These were screened against the eligibility criteria and 602 were excluded, with 357 trials included (Fig. 1). The main reasons for exclusion were the purpose of the trial (e.g., prevention), date of registration (e.g., after 31 December 2021) and trial design (e.g., quasi-randomised).

### Study general characteristics

Of the 357 included studies, 192 (53.8%) were in the hydroxychloroquine group, 108 (30.2%) were in the corticosteroid group and 57 (16%) were in the vitamin D group (Table 1). Of the trials that stated the study design, parallel assignment was used in 309 (94.5%) of the trials, with cross-over, sequential and factorial study designs making up the remaining 18 (5.5%). Of the 320 trials that stated the phase of the trial, 170 (53%) were phase 3 trials, 76 (24%) of the trials were labelled as phase 2 and 67 (21%) were labelled as phase 4. Seven (2%) trials were classified as phase 0 or 1 or N/A, none of which had results found in the public domain. Of the registered trials that stated the country of origin, 20 (5.8%) were multinational. Three quarters of the registered trials (268, 75.1%) had two randomised groups and 89 (24.9%) of the trials had more than two. A placebo control group was used in 107 (30%) of the trials. An email address of a contact person or lead researcher was available for 204 (57%) trials. Medical centres were the primary sponsor for 128 (35.9%) of the trials, with academic facilities for 115 (32.2%), pharmaceutical companies for 15 (4.2%) and government for 18 (5%).

**Table 1:** Trial characteristics (design)

Characteristics, n (%)	HCQ	Steroid	Vit D	Total, n (%)
	**192**	**108**	**57**	**357**
**Study design info provided**	**172**	**103**	**52**	**327**
Parallel	163 (94.8)	99 (96)	47 (90.4)	309 (94.5)
Factorial	7 (4)	2 (2)	5 (9.6)	14 (4.3)
Other	2 (1.2)	2 (2)	0 (0)	4 (1.2)
**Phase of trial info provided**	**182**	**95**	**43**	**320**
Phase 0 or 1	4 (2.2)	2 (2.1)	1 (2.3)	7 (2.2)
Phase 2 or 1–2	59 (26.9)	16 (16.8)	11 (25.6)	76 (23.8)
Phase 3 or 2–3	93 (51.1)	57 (60)	20 (46.5)	170 (53.1)
Phase 4 or 3–4	36 (19.8)	20 (21.1)	11 (25.6)	67 (20.9)
**Country info provided**	**184**	**102**	**57**	**343**
Multinational	11 (6)	7 (6.9)	2 (3.5)	20 (5.8)
**No. arms info provided**	**192**	**108**	**57**	**357**
> 2 randomised groups	63 (32.8)	15 (13.9)	11 (19.3)	89 (24.9)
**Control info provided**	**192**	**108**	**57**	**357**
Placebo control	62 (32.3)	15 (13.9)	30 (52.6)	107 (29.9)
**Primary sponsor info provided**	**192**	**108**	**57**	**357**
Academic	52 (27.1)	41 (38)	22 (38.6)	115 (32.2)
Research institute	18 (9.4)	15 (13.9)	8 (14)	41 (11.5)
Governmental	13 (6.8)	2 (1.8)	3 (5.3)	18 (5)
Medical centre	80 (41.6)	35 (32.4)	13 (22.8)	128 (35.9)
Pharmaceutical	8 (4.2)	4 (3.7)	3 (5.3)	15 (4.2)
Other	19 (9.9)	10 (9.3)	8 (14)	37 (10.4)
No sponsor	2 (1)	1 (0.9)	0 (0)	3 (0.8)
**Email contact info provided**	**192**	**108**	**57**	**357**
Email contact available	98 (51)	70 (64.8)	36 (63.2)	204 (57.1)

### Design characteristics

Of the trials that stated their target sample size, 155 (44.3%) were small trials (≤ 100 patients), 105 (30%) medium sized trials (101 to 300 patients) and 90 (25.7%) large trials (> 300 patients) (Table 2). Of the 297 trials that stated if blinding was applied, 138 (46.5%) used blinding. Of these, 135 described the level of blinding and 108 (80%) of those reported that they were double, triple or quadruple blinded. Most trial records failed to report allocation concealment, however, almost all (33, 94.3%) of those that did mention allocation concealment reported it had been applied. Ethics approval status was reported for 157 (44%) trials, and of those, one reported that they did not have ethics approval.

Trial status, according to the primary registries, was 28 (7.8%) not stated or unknown, 159 (44.5%) ongoing, 72 (20.2%) completed and 98 (27.5%) withdrawn/terminated (Table 2). However, the registry entry for many of the trials that were shown as “ongoing” had not been updated for more than 12 months. Of the 98 trials in the withdrawn/terminated category, 12 were listed as suspended, 28 as withdrawn and 58 as terminated/prematurely ended. Reasons for termination varied but included recruitment challenges and emerging evidence that was not suggestive of efficacy of the interventional drug.

**Table 2:** Trial characteristics (methods)

Characteristics, n (%)	HCQ	Steroid	Vit D	Total n (%)
**Target sample size info provided**	**187**	**106**	**57**	**350**
Small (≤ 100)	83 (44.4)	48 (45.3)	24 (42.1)	155 (44.3)
Medium (101 to 300)	54 (28.9)	33 (31.1)	18 (31.6)	105 (30)
Large (> 300)	50 (26.7)	25 (23.6)	15 (26.3)	90 (25.7)
**Blinding info provided**	**170**	**108**	**19**	**297**
Blinding	82 (48.2)	40 (37.)	16 (84.2)	138 (46.5)
**Blinding description info provided**	**79**	**40**	**16**	**135**
Single	14 (17.7)	11 (27.5)	2 (12.5)	27 (20)
Double	32 (40.5)	19 (47.5)	8 (50)	59 (43.7)
Triple/Quadruple	33 (41.8)	10 (25)	6 (37.5)	49 (36.3)
**Allocation concealment info provided**	**12**	**12**	**11**	**35**
Allocation concealment	12 (100)	10 (83.3)	11 (100)	33 (94.3)
**Ethics info provided**	**76**	**58**	**23**	**157**
Ethics approval	75 (98.7)	58 (100)	23 (100)	156 (99.4)
**Trial status info provided**	**192**	**108**	**57**	**357**
Not stated/Unknown	18 (9.4)	6 (5.6)	4 (7)	28 (7.8)
Ongoing	70 (36.5)	59 (54.6)	30 (52.6)	159 (44.5)
Completed	34 (17.7)	20 (18.5)	18 (31.6)	72 (20.2)
Withdrawn/Terminated	70 (36.4)	23 (21.3)	5 (8.8)	98 (27.5)

### Outcome

Our primary outcome of interest for this study was the publication status of the registered trials. Our public platform search identified 107 (30%) of the trials had been published in a peer reviewed journal or had made their results publicly available in another way, while 250 (70%) of the registered trials had not (Table 3). When assessing the extent of the publication, we found that 77 (21.6%) had been published in a peer-reviewed journal and a further 13 (3.6%) were found on preprint servers. Twelve ICTRP entries mentioned that results were available but had not shared these results. Three studies were published as a research letter and two in a retraction notice.

**Table 3:** Outcome - Trial publication status

Publication status, n (%)	HCQ	Steroid	Vit D	Total
Not published	135 (70.3)	78 (72.2)	37 (65)	250 (70)
Peer-reviewed	40 (20.8)	23 (21.3)	14 (24.6)	77 (21.6)
Preprint	6 (3.1)	4 (3.7)	3 (5.2)	13 (3.6)
Results on ICTRP	9 (4.8)	1 (0.9)	2 (3.5)	12 (3.4)
Research letter	1 (0.5)	2 (1.9)	0 (0)	3 (0.8)
Retracted	1 (0.5)	0 (0)	1 (1.7)	2 (0.6)

The univariate analyses for the association of trial characteristics and publication status found few factors that were statistically significant (Table 4). A trial with a large target sample size (> 300) had almost twice the odds (OR: 1.85, 95%CI: 1.05 to 3.25) to be published as one with smaller target sample sizes, while trials with a medical centre as the primary sponsor had about half the odds (OR: 0.46, 95%CI: 0.26 to 0.82) to be published as those with an academic primary sponsor. Trials that were multinational, phase 3 or 4, placebo controlled or used blinding had an odds ratio suggesting an increase in publication likelihood, but their accompanying confidence intervals included no difference (Table 4). Sensitivity analysis limiting the subgroup of trials labelled “complete” or “terminated” revealed no significant differences in the direction or significance of factors when compared to the full dataset of trials.

The multiple logistic regression analysis showed that target sample size was a significant positive predictor of publication. Medium target sample size doubled the odds of publication compared to small target sample size (aOR: 2.1, 95% CI: 1.08 to 4.1) and large target sample sizes almost tripled the odds (aOR: 2.75, 95% CI: 1.35 to 5.62). The model also showed that, compared to being sponsored by an academic institution, being sponsored by a medical centre (aOR: 0.31, 95%CI: 0.12 to 0.77), the government (aOR: 0.28, 95%CI: 0.07 to 0.89) or a research institute (aOR: 0.38, 95%CI: 0.15 to 0.95) as well as having an email address on ICTRP (aOR: 0.31, 95%CI: 0.17 to 0.57) were statistically significant negative predictors for publication.

**Table 4:** Multivariable logistic regression analysis for publication status

Characteristics	Crude OR	95% CI	Adjusted OR	95% CI
Drug				
Vitamin D	1.00	Ref		
Corticosteroid	0.71	0.36–1.42		
Hydroxychloroquine	0.78	0.42–1.46		
Parallel study design	0.67	0.25–1.77		
Phase 3 or 4	1.47	0.82–2.63	1.28	0.92–1.78
Multinational	1.88	0.75–4.68	0.96	0.32–2.86
> 2 randomised groups	0.71	0.41–1.22	0.56	0.28–1.09
Placebo controlled	1.54	0.95–2.49	1.34	0.75–2.39
Primary sponsor				
Academic	1.00	Ref		
Research institute	0.56	0.25–1.26	0.38	0.15–0.95
Government	0.67	0.22–2.01	0.26	0.07 to 0.89
Medical centre	0.46	0.26–0.82	0.28	0.14–0.56
Pharmaceutical	0.87	0.28–2.71	0.96	0.29–3.21
Other	1.4	0.69–2.95	0.85	0.36–2.04
Email address on ICTRP	0.49	0.31–0.78	0.32	0.18–0.58
Target sample size				
≤ 100	1.00	Ref		
> 100 ≤ 300	1.5	0.88–2.65	2.1	1.08–4.1
> 300	1.85	1.05–3.25	2.75	1.35–5.62
Blinding	1.31	0.8 – 2.14		

We found that eight chloroquine or hydroxychloroquine treatment trials had been registered after the publication of the Cochrane Review on their use for prevention and treatment of COVID-19 in February 2021 [10]. For all eight, the status of the trial in the primary registries as of 20 October 2022 was either recruiting, active (recruiting completed) or completed. Results of three of these trials were found in the public domain search, one as a peer-reviewed article and two on preprint servers. In regard to corticosteroids, the Cochrane Review, despite finding positive outcomes for the use of corticosteroids, urged researchers to continue with research in this area specifically encouraging good quality evidence for specific subgroups of disease severity [19]. Therefore, we did not assess whether the nine corticosteroid trials registered after the publication of the Cochrane Review on corticosteroids might represent research waste.

## Discussion

Among the characteristics we examined, we found that an increased odds of publication was associated with medium or large target sample size; and that having a medical centre, the government or a research institute as the primary sponsor was associated with lower odds of publication. Similarly, in other areas of health research, intervention type and particular types of study sponsors, specifically a pharmaceutical company, have been found to significantly influence publication status [23]. Although it has been shown that some trial characteristics influence the quality and risk of bias for a trial, such as the use of blinding and allocation concealment, these characteristics were not found to be associated with trial publication status in our study [24]. The assumption that a multinational trial or one with more than two randomised groups would have more key role-players and therefore more support and a higher chance of being published was also not demonstrated by this study.

In other health fields, such as oncology, the publication of non-significant results is a challenge, even for bigger trials [25]. At the height of the pandemic, journals were inundated with submissions about COVID-19 and were having to process manuscripts that might be reporting research that would make little or no contribution to the evidence base because of the rapidity with which some areas were developing, which might have been particularly problematic for the large number of small trials as the results of larger, more definitive trials became available.

As far as we know, this study is the first to compare publication status of registered randomised trials of treatments for COVID-19 with trial characteristics revealing a concerningly low publication rate of COVID-19 trials. In other areas of health, similar trends were seen, for example one study reported that less than half of trials registered on *clinicaltrials.gov* had been published, while another reported that only 28.6% of the registered trials were published within 24 months of trial completion [26, 27]. Our results shed some light on this, as larger trials were more likely to be published, and potentially appropriately funded to publish and afford publication costs, compared to smaller singe centre trials. More precise trial sponsor and funding data is required in trial registries as these are potentially important predictors of research waste and necessary information for researchers, funders and policy makers.

On ICTRP, only 20 studies reported that their results were available, and of these seven had published in a peer-reviewed journal. However, our search found a total of 77 (22%) of the trials in our sample had been published in a peer-reviewed journal. Trials classified as “completed” accounted for 72 (20%) of the eligible trials in this study and we found that 25 of these had been published in peer-reviewed journals and seven had shared their results publicly in some other way, leaving 40 “completed” trials with unshared results. Furthermore, we found that 39 of the 77 studies that were still classified as “ongoing” on the primary registry had been published in a peer-reviewed journal. In some cases, this was due to the publication of interim results of a trial or partial results for one treatment group in the case of platform trials, but this seems unlikely for all these 39 trials, and we expect that many have completed without updating their registry entry. This reinforces that there is a problem with a lack of updating of the trial registry by researchers.

The “ongoing” category made up 44% of the trials in our study, and included recruiting trials, trials that began towards the end of our timeframe for eligible registrations (31 December 2021), trials that began earlier but were taking longer and trials that may have been completed but without an update to the registry entry. Some of these trial entries have not been updated for over 12 months. It is possible that an important proportion of these are in fact no longer ongoing. This highlights the importance of researchers updating the trial registries on a regular basis to ensure the status of their trial is transparent and their results known, especially if the trial is no longer ongoing.

A limitation of our study is with the search for publications. For instance, if the title used for a trial in its registry entry was not used in its publication or the published article did not include the trial identifier, it would not have been found during our search. Furthermore, a search beyond Google Scholar and PubMed might have found more publications. It would also have helped those searching for trial results if the published reports of all trials included the trial’s registration ID, which would help to reduce waste by making it easier for users to find the research. In addition to the extent of our search, a further constraint is that only one author performed the search, screening and data extraction.

Another potential limitation of our study is that we collected most of the data from ICTRP. As ICTRP draws its data from multiple registries it creates an entry for each unique trial ID. If a research team register their trial on more than one registry and do not list the same title and research team, this may lead to duplicates which we were unable to rule out. Each entry was therefore treated as a separate trial. Moreover, some loss of data detail could have occurred because of the information transfer from the primary registry to ICTRP. However, when we reviewed primary registries for key variables, we found gaps in some of these. Trial registries vary in the registration information and updating requirements which has an impact of the quality and availability of this data and possibly on publication. We recommend that trial registries have firmer guidelines and updating policies and that trialists are specific as possible when uploading and updating information in the registry.

## Conclusions

We performed a meta-epidemiological cohort study to describe the characteristics and publication status of registered randomised trials of the use of corticosteroids, hydroxychloroquine, and vitamin D to treat patients with COVID-19 and to analyse the association between trial characteristics and publication status. Our findings suggest that target sample size of above 100 patients is an important predictor of publication. This may be related to other factors such as funding or the institutions involved in a trial but highlights the need for researchers to collaborate on large, definitive trials to increase their chances of publication and making an important contribution to the evidence base. We found that a large proportion of registered trials have not yet made their results publicly available. Even for trials not published, if the results are not made publicly available at minimum, this amounts to research waste. Our findings also identified a discrepancy between the trial’s status of some trials in the registry and their publication status, with publications for some of those marked as “ongoing” on the registry having been published and likely to be completed.

In order to reduce research waste and to ensure that the rush to do research during the COVID-19 pandemic does not lead to the “equivalent of the boxes of useless aid supplies that end up rotting away on runways and in warehouses after large scale disasters and humanitarian emergencies” [3] we recommend that trial registries are updated more frequently and that results of trials are made public. We also recommend that further investigations are done to assess for factors contributing to publication status and research waste and that these factors are carefully considered before studies are designed, funded, granted ethical or regulatory approval and initiated both in further emergencies such as the COVID-19 pandemic but also in health care in more normal times.

### Electronic supplementary material

Below is the link to the electronic supplementary material.

Supplementary Material 1

Supplementary Material 2

## Data Availability

The datasets used and/or analysed during the current study are available from the corresponding author on reasonable request.

## List of abbreviations

WHO: World Health Organization
ICTRP: International Clinical Trial Registry Platform
ICMJE: International Committee of Medical Journal Editors
HCQ: Hydroxychloroquine
Vit D: Vitamin D
