# Supplementary material for: Exploring trial publication and research waste in COVID-19 randomised trials of hydroxychloroquine, corticosteroids, and vitamin D: a meta-epidemiological cohort study
Source: BMC Med Res Methodol. 2024 Jan 23;24:19. doi: 10.1186/s12874-023-02110-4 (PMC10804507; doi:10.1186/s12874-023-02110-4)
Supplement: Supplementary file 1 — Supplementary Material 1 [file 12874_2023_2110_MOESM1_ESM.docx]

Additional file 1

Search strategy:

Hydroxychloroquine OR Chloroquine OR Hydroxychloroquin* OR Hydroxychloroquin* OR chloroquin*

dexamethason* OR "BB 1101" OR decadron OR hexadrol OR fortecortin OR dexameth OR dexone OR hexadecadrol OR desamethason* OR ozurdex OR deronil OR baycuten OR aacidexam OR spersadex OR dexacortal OR gammacorten OR visumetazon* OR adexone OR "Alba‐Dex" OR cortidexason OR decacort OR decadrol OR dectancyl OR desameton OR loverine OR millicorten OR orgadrone OR alin OR auxiloson OR cortisumman OR decalix OR decameth OR decasone OR dekacort OR deltafluorene OR "Dexa‐Mamallet" OR dexafluorene OR dexalocal OR dexamecortin OR dexamonozon OR dexapos OR dexinoral OR fluorodelta OR lokalison OR methylfluorprednisolon* OR mymethason* OR "Dexa‐Rhinosan" OR "Dexa‐Scheroson" OR "Dexa‐sine" OR dexacortin OR dexafarma OR dinormon OR baycadron OR "Aeroseb‐Dex" OR Maxidex OR Dextenza OR dexasone OR dexpak

vitamin d" OR "vitamind" OR "vitamin d3" OR "vitamin d2" OR "hydroxyvitamin d" OR "dihydroxyvitamin d" OR cholecalciferol* OR colecalcifer* OR calciferol* OR calciol* OR calcidiol* OR calcitriol* OR calcifediol* OR calciferol* OR ercalcidiol* OR ercalcitriol* OR ergocalciferol* OR doxercalciferol* OR colecalciferol* OR paricalcitol* OR alphacalcidol* OR dihydrotachysterol*
