# Supplementary material for: Exploring trial publication and research waste in COVID-19 randomised trials of hydroxychloroquine, corticosteroids, and vitamin D: a meta-epidemiological cohort study
Source: BMC Med Res Methodol. 2024 Jan 23;24:19. doi: 10.1186/s12874-023-02110-4 (PMC10804507; doi:10.1186/s12874-023-02110-4)
Supplement: Supplementary file 2 — Supplementary Material 2 [file 12874_2023_2110_MOESM2_ESM.docx]

Additional file 2

List of trial characteristics extracted:

- Drug of interest (categorical)
- Study design (categorical)
  - Parallel
  - Factorial
  - Cross-over/Sequential
- Phase of trial (ordinal)
  - Phase 0/1
  - Phase 2 /1-2
  - Phase 3 / 2-3
  - Phase 4 / 3-4
- Multinational (binary)
- Multi-arm (binary)
- Placebo controlled (binary)
- Primary sponsor (categorical)
  - Academic
  - Research institute
  - Governmental
  - Medical Centre
  - Pharmaceutical
  - Other
  - No sponsor
- Email contact on ICTRP (binary)
- Target sample size (continuous)
- Target sample size (ordinal)
  - Small (≤100)
  - Moderate (>100 ≤300)
  - Large (>300)
- Blinding (binary)
- Blinding description (categorical)
  - Single
  - Double
  - Triple/ Quadruple
- Allocation concealment (binary)
- Ethics approval (binary)
- Trial status (ordinal)
  - Not started/ Unknown
  - Ongoing
  - Completed
  - Withdrawn/Terminated
- Publication status
  - Not published
  - Published
    - Results availability mentioned on ICTRP
    - Other
    - Preprint
    - Peer-reviewed journal
